# Compound Absorption in Polymer Devices Impairs the Translatability of Preclinical Safety Assessments

**DOI:** 10.1002/adhm.202303561

**Published:** 2023-12-10

**Authors:** Aurino M. Kemas, Reza Zandi Shafagh, Nayere Taebnia, Maurice Michel, Lena Preiss, Ute Hofmann, Volker M. Lauschke

**Affiliations:** ^1^ Department of Physiology and Pharmacology Karolinska Institutet Stockholm 17177 Sweden; ^2^ Dr. Margarete Fischer‐Bosch Institute of Clinical Pharmacology 70376 Stuttgart Germany; ^3^ University of Tuebingen 72074 Tuebingen Germany; ^4^ Division of Micro‐ and Nanosystems KTH Royal Institute of Technology Stockholm 10044 Sweden; ^5^ Department of Oncology and Pathology Science for Life Laboratory Karolinska Institutet Stockholm 17165 Sweden; ^6^ Department of Drug Metabolism and Pharmacokinetics (DMPK) Merck KGaA 64293 Darmstadt Germany

**Keywords:** drug development, logP, microphysiological system, polymer‐drug interaction, small molecule absorption

## Abstract

Organotypic and microphysiological systems (MPS) that can emulate the molecular phenotype and function of human tissues, such as liver, are increasingly used in preclinical drug development. However, despite their improved predictivity, drug development success rates have remained low with most compounds failing in clinical phases despite promising preclinical data. Here, it is tested whether absorption of small molecules to polymers commonly used for MPS fabrication can impact preclinical pharmacological and toxicological assessments and contribute to the high clinical failure rates. To this end, identical devices are fabricated from eight different MPS polymers and absorption of prototypic compounds with different physicochemical properties are analyzed. It is found that overall absorption is primarily driven by compound hydrophobicity and the number of rotatable bonds. However, absorption can differ by >1000‐fold between polymers with polydimethyl siloxane (PDMS) being most absorptive, whereas polytetrafluoroethylene (PTFE) and thiol‐ene epoxy (TEE) absorbed the least. Strikingly, organotypic primary human liver cultures successfully flagged hydrophobic hepatotoxins in lowly absorbing TEE devices at therapeutically relevant concentrations, whereas isogenic cultures in PDMS devices are resistant, resulting in false negative safety signals. Combined, these results can guide the selection of MPS materials and facilitate the development of preclinical assays with improved translatability.

## Graphical abstract

Identical microdevices are fabricated from eight different polymers and it is found that absorption of prototypic compounds with different physicochemical properties differs by >1000‐fold between materials. Using 3D human liver cultures, it is shown that minimizing absorption can improve the accuracy of pharmacological or toxicological testing. These results have important implications for improving preclinical assay accuracy in drug development.

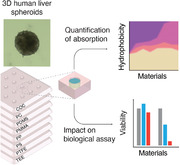

## 1 Introduction

Success rates of drug development projects remain low despite major advances in enabling technologies, such as combinatorial chemistry, gene editing, multidimensional omics profiling, and phenotypic screening. Compound toxicity is among the most common reasons for the discontinuation of compounds nominated for further development in phase I or phase II,^[^ ^1^, ^2^ ^]^ and the liver is among the most commonly responsible organ systems.^[^ ^3^ ^]^ Importantly, the quality of preclinical safety assessments is strongly correlated with the risk of safety‐related project closures^[^ ^2^ ^]^ and success rates remain stably low, despite a steady increase in resources committed to drug development programs.^[^ ^4^ ^]^ Development of methods, tools, and assays that improve the prediction of clinical compound toxicity is thus an area of major importance for drug developers and the pharmaceutical industry.^[^ ^5^ ^]^

The translatability of in vitro findings varies drastically among cell culture systems. Hepatic cell lines and 2D cultures of primary human hepatocytes (PHH) have been predominantly used to predict liver toxicity. However, molecular phenotypes and functions of cell lines do not closely resemble mature human hepatocytes.^[^ ^6^, ^7^, ^8^ ^]^ Furthermore, PHHs rapidly deteriorate within a few hours when cultured in conventional 2D monolayers.^[^ ^9^ ^]^ As a consequence of this dedifferentiation, the predictive power of such culture models remains overall low.^[^ ^10^ ^]^ In recent years, it has become increasingly clear that more advanced culture systems, such as micropatterned cocultures,^[^ ^11^, ^12^ ^]^ sandwich cultures,^[^ ^13^, ^14^ ^]^ or 3D spheroids^[^ ^15^, ^16^ ^]^ can maintain hepatic molecular signatures and liver functions for up to 5 weeks. Likely because of these improved phenotypes, such advanced culture methods more closely recapitulate toxicity mechanisms^[^ ^17^ ^]^ and have improved sensitivity and selectivity to predict clinical drug‐induced liver injury.^[^ ^18^, ^19^, ^20^ ^]^ Microphysiological systems (MPS) which combine organotypic culture methods with microfluidic perfusion can further extend the physiological relevance of cell models by adding hemodynamic control and the possibility to emulate hepatic zonation.^[^ ^21^ ^]^ Moreover, previous studies showed that perfusion provides minor but measurable improvements of hepatic cell phenotypes^[^ ^22^, ^23^ ^]^ and allows for monolithic integration with electrical, electrochemical, and optical sensors for real‐time on‐chip monitoring of cell impedance,^[^ ^24^ ^]^ shear stress,^[^ ^25^ ^]^ pH, and temperature.^[^ ^26^ ^]^

Besides cell models, the material of the culture vessel can directly impact assay performance.^[^ ^27^ ^]^ Unlike cell culture plates for static cultures, which most commonly consist of polystyrene (PS), the choice of material for the development of MPS is typically guided by the ease of fabrication and the required investment in microengineering infrastructure. Based on these considerations, poly(dimethyl siloxane) (PDMS) has emerged as the most widely used material for microfluidic device fabrication and remains the primary material of choice for research purposes and, more recently, for use in preclinical drug testing.^[^ ^28^ ^]^ However, it has become clear that PDMS is highly absorptive of hydrophobic small molecules, which complicates pharmacological and toxicological studies.^[^ ^29^, ^30^ ^]^ While PDMS is most widely used, other biocompatible thermoplastic and thermosetting materials, including poly(methyl methacrylate) (PMMA),^[^ ^31^ ^]^ polycarbonate (PC),^[^ ^32^ ^]^ cyclic olefin copolymer (COC),^[^ ^33^ ^]^ and thiol‐ene epoxy (TEE)^[^ ^34^ ^]^ have been presented and successfully applied to the development of microfluidic devices.

Here, we provide the first quantitative head‐to‐head comparison of the absorptive capacity of polymers (PDMS, PS, PMMA, COC, TEE, PC, polypropylene[PP], and polytetrafluoroethylene[PTFE]) commonly used for cell culture and preclinical drug testing. Using these materials, we fabricated identical devices and measured the absorption kinetics of prototypic drugs with different physicochemical properties using liquid chromatography with tandem mass spectroscopy. We find that the extent of absorption differs by >1000‐fold for hydrophobic drugs across polymers with PDMS being the most absorptive, while TEE and PTFE absorbed the least. Most of the variability in drug absorption across polymers was explained by compound hydrophobicity (*logP*) with additional contributions from the number of hydrogen donors and rotatable bonds. In contrast, the hydrophobicity of the platform material was not associated with drug absorption. Importantly, by comparing compound preclinical safety assessments in highly and lowly absorbing devices using isogenic organotypic 3D human liver cultures, we show that reduced drug absorption directly translates into increased assay sensitivity, thereby improving outcomes of in vitro toxicity tests. Combined, our results demonstrate the importance of platform material for pharmacological and toxicological test systems, identify key physicochemical properties that predict the extent of compound absorption and provide guidance for the selection of platform material to optimize for preclinical drug development.

## 2 Results

### 2.1 Commonly Used Polymers Drastically Differ in their Absorption Profiles

**Figure 1:**
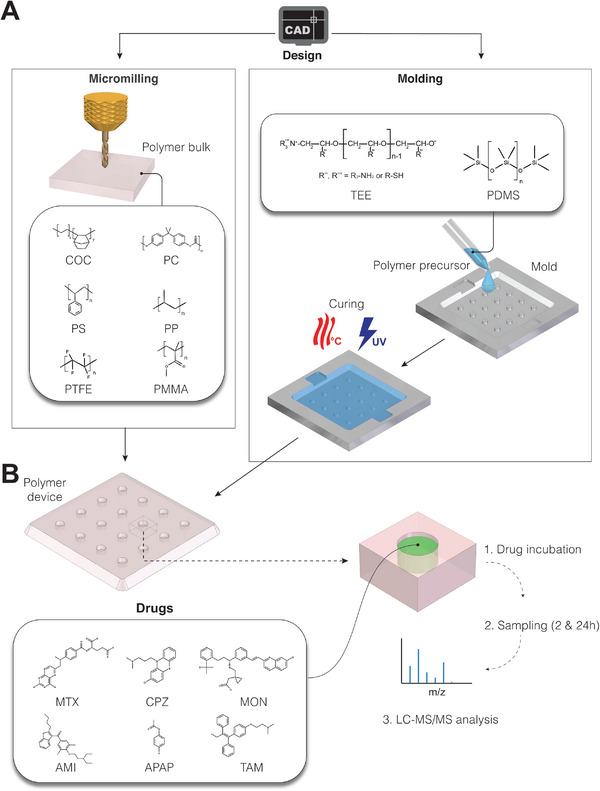
Measuring the absorption of prototypic small molecules in commonly used cell culture polymers. A) The devices used in this study were fabricated by micromilling (for COC, PC, PS, PP, PMMA, and PTFE) and molding (for PDMS and TEE), resulting in devices with identical surface‐to‐volume ratio (see Methods for fabrication details and Figure S1 for device dimension, Supporting Information). B) Compounds with different physicochemical properties were incubated in the respective devices and free compound concentrations were measured by liquid chromatography with tandem mass spectroscopy (LC‐MS/MS) after 2 and 24 h. The free compound concentrations were compared to input concentrations to determine the absorption. AMI = amiodarone; APAP = acetaminophen; COC = cyclic olefin co‐polymer, CPZ = chlorpromazine; MON = montelukast; MTX = methotrexate; PC = polycarbonate; PDMS = polydimethylsiloxane; PMMA = polymethylmethacrylate; PP = polypropylene; PS = polystyrene; PTFE = polytetrafluoroethylene; TAM = tamoxifen; TEE = thiol‐ene epoxy.

To systematically evaluate and benchmark drug absorption of thermoplastic and thermosetting polymers, we selected eight materials commonly used in microfluidic applications. Specifically, we selected PDMS, PS, COC, PC, PMMA, PTFE, PP, and TEE. We then fabricated devices with identical geometries and dimensions from all these materials using both micromilling and casting techniques (**Figure** **1**A). Specifically, we designed the shape and size of our device compartments to resemble wells in standard 96‐well plates, which facilitates comparisons to the many static cultures in the literature that use this format. While MPS designs are substantially more heterogeneous, similar compartment sizes and shapes have also been used in some perfused systems.^[^ ^35^ ^]^ To assess absorption, we selected prototypic compounds with widely differing physicochemical properties, such as size, hydrophobicity, topological polar surface area, number of hydrogen donors and acceptors and number of rotational bonds (**Table** **1**). We preferentially selected known hepatotoxins to be able to directly evaluate the impact of absorption on typical preclinical assay readouts. Loss of compound was quantified by measuring free concentrations of the selected compounds before and after incubation across all polymer devices by LC‐MS/MS (Figure 1B).

**Table 1:** Compounds used in this study and their physicochemical properties. AMI = amiodarone; APAP = acetaminophen; CPZ = chlorpromazine; HAC = hydrogen acceptor count; HDC = hydrogen donor count; *logP* = octanol/water partition coefficient in log_10_; *logS* = water solubility in log_10_; MON = montelukast; MTX = methotrexate; MM1‐MM3 = APAP derivates 1–3: 4‐methoxyaniline, 4‐(difluoromethoxy)‐aniline, and *N*‐methyl‐4‐(trifluoromethoxy)aniline, respectively; RBC = rotatable bond count; TAM = tamoxifen; TPSA = topological polar surface area.

Compounds	Physicochemical features/properties						
	*M*_w_ [g mol^−1^]	LogP	LogS	TPSA [Å^2^]	HDC	HAC	RBC
TAM	371.5	5.93	−5.56	12.47	0	2	8
CPZ	355.3	5.18	−4.88	31.8	1	2	4
MON	608.2	8	−8.3	98.6	1	5	12
AMI	681.8	7.24	−5.1	42.68	1	4	11
APAP	151.2	0.51	−1.6	49.33	2	2	1
MTX	454.4	‐0.91	−3.7	205.92	6	12	9
MM1	123.16	1.01	−0.73	35.26	1	2	1
MM2	159.13	1.7	−1.2	35.26	1	4	2
MM3	191.15	3.32	−2.74	21.26	1	5	2

**Figure 2:**
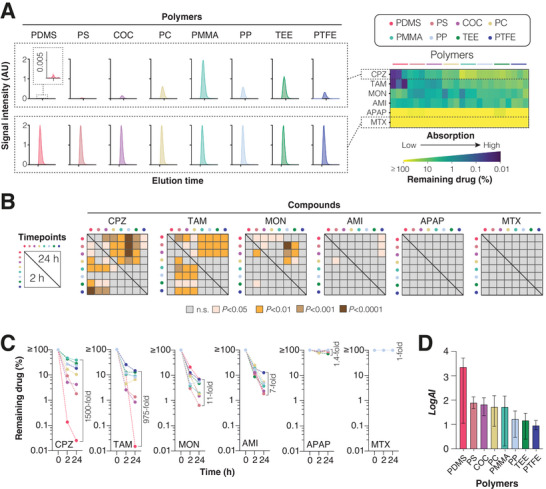
The choice of polymers has a major impact on the concentrations of free compound. A) Left: Mass spectrometry peaks of free chlorpromazine (CPZ) and methotrexate (MTX). Note that the size of CPZ peaks differs drastically between polymers whereas MTX absorption was negligible in any material tested. Right: Heatmap visualization of total absorption after 24 h incubation across polymers. For each polymer three independent replicate measurements are shown. B) Matrices showing pairwise comparisons of absorption rates between polymers after 2 and 24 h. Differences were considered significant for a given compound between two polymers, if absorption differed by >30%. Benjamini–Hochberg correction with a false discovery rate (FDR) of 10% was used to account for multiple testing. C) Absorption kinetics are shown for each compound across polymer devices. Each dot represents the median value from 3 independent measurements in each polymer device. Fold differences between the highest and lowest absorbing polymers are indicated. D) Absorption indices (AI; see Methods for definition) are shown for each polymer with higher *logAI* indicating higher absorption into the polymer bulks. Each bar represents the median ± range of *logAI* across all compounds with nonzero absorption. In each polymer, the *logAI* for each compound was represented by the average from measurements in three independent devices. AMI = amiodarone; APAP = acetaminophen; COC = cyclic olefin copolymer; CPZ = chlorpromazine; MON = montelukast; PC = polycarbonate; PDMS = polydimethylsiloxane; PMMA = polymethylmethacrylate; PP = polypropylene; PS = polystyrene; PTFE = polytetrafluoroethylene; TAM = tamoxifen; TEE = thiol‐ene epoxy.

Our analysis revealed that the loss of free compound differed drastically between polymers and drugs (**Figure** **2**A,B). Whereas compound concentrations in solution remained stable for methotrexate and acetaminophen in any material, chlorpromazine, tamoxifen, montelukast, and amiodarone exhibited significant loss of compound as early as after 2 h of incubation. Measured concentrations after incubations can decrease due to evaporation, degradation, surface adsorption or absorption into the bulk of the polymer. Evaporation was <6% v/v for each polymer (Figure S2, Supporting Information) and we did not observe any degradation product peaks in the LC‐MS/MS spectra. We thus conclude that the observed loss of compound is driven at least predominantly by adsorptive and absorptive processes that we jointly refer to as absorption henceforth.

Strikingly, for a given compound, absorption differed drastically between platform materials (Figure 2C). While more than 99% of chlorpromazine partitioned into the PDMS bulk after 24 h incubation, absorption rates in PMMA and TEE were around 1500 times lower (*P* < 0.0001). Similar results were obtained for tamoxifen where PDMS was 975‐fold more absorptive than PTFE and TEE. In contrast, montelukast and amiodarone displayed only relatively minor differences (7‐ to 11‐fold) between polymers while acetaminophen and methotrexate did not absorb in any material. At 37 °C there was an overall trend of increased absorption compared to 25 °C (Figure S3, Supporting Information). These effects however were similar in highly (PDMS) and lowly absorbing materials (TEE) and thus did not majorly affect the interpretation of absorption differences between polymers. Overall, the absorption index (*logAI*), defined as the logarithm of the ratio of compound absorbed into the polymer bulk versus the compound that remained in solution, was highest for PDMS and lowest for PTFE and TEE (Figure 2D). Combined, these data suggest that the choice of material can have major impacts on free compound concentrations.

### 2.2 Computational Modeling of Drug Absorption Recapitulates Experimental Data

**Figure 3:**
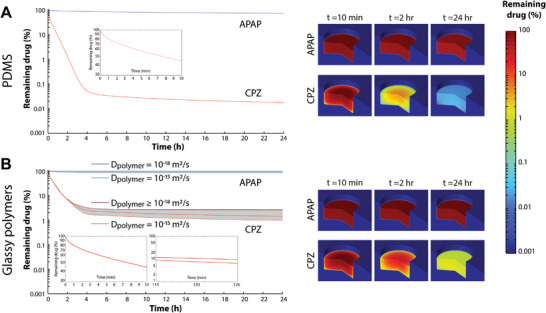
Time‐dependent modeling of drug concentrations in rubbery and glassy polymers. Simulation results for drug absorption within PDMS A) and glassy polymers B) with in‐polymer diffusion coefficients (*D*_polymer_) from 10^−18^ to 10^−15^ m^2^ s^−1^ over time. Left: Absorption profiles are shown for acetaminophen (APAP) and chlorpromazine (CPZ) as examples of lowly and highly absorbing drugs, respectively. Right: 3D heat maps of the concentration of model drugs (with low to high absorptivity) after 10 min, 2, and 24 h.

To further understand absorption in the devices, we conducted 3D simulations using COMSOL modeling, considering a spectrum of diffusion coefficients (*D*_polymer_; see the Experimental Section for details). Importantly, time‐dependent simulation closely approximated the partitioning dynamics of both acetaminophen and chlorpromazine as prototypic examples of nonabsorbing and absorbing small molecules, respectively. The octanol:water partition coefficient was used as an approximate substitute for PDMS:water in our simulation study.^[^ ^36^ ^]^ Notably, this approximation might introduce inaccuracies due to the differences in the physicochemical properties of octanol and the actual polymer and precise numerical values for specific compound‐polymer pairs may vary to some extent from these estimates. In PDMS, acetaminophen was only lowly absorbed, whereas chlorpromazine exhibited high absorption with free concentrations well below 1% already after 2 h (**Figure** **3**A). While acetaminophen profiles in glassy polymers were identical to PDMS, the remaining concentrations of chlorpromazine were significantly higher (Figure 3B). However, modeled free chlorpromazine concentrations were lower than experimentally determined. This underestimation can likely be attributed to temperature gradients within the experimental setup that result in nondiffusive transport mechanisms. To test this hypothesis, we modeled effects of an arbitrary temperature difference between the bulk liquid and the container wall of 2 °C (Figure S4A, Supporting Information). Notably, introduction of this gradient corrected the underestimation and increased alignment with experimental data.

In‐polymer diffusion coefficients had only modest impacts on long‐term drug absorption, suggesting that additional polymer properties modulate absorption. Absorption in either polymer was very rapid with >50% of compound loss already after 10 min but differed by orders of magnitude after 2 and 24 h, aligning with experimental data. Interestingly, increasing compound logP affected neither the slope of the initial absorption phase nor of long‐term equilibration (Figure S4B, Supporting Information). Rather, logP increased the extent of free compound loss by prolonging the initial absorption phase. Combined, these results thus corroborate that, contingent upon partition coefficient and polymer diffusivity, different polymer devices can rapidly yield markedly different exposure concentrations.

### 2.3 Drug Absorption Can Bias the Outcome of Toxicity Testing

**Figure 4:**
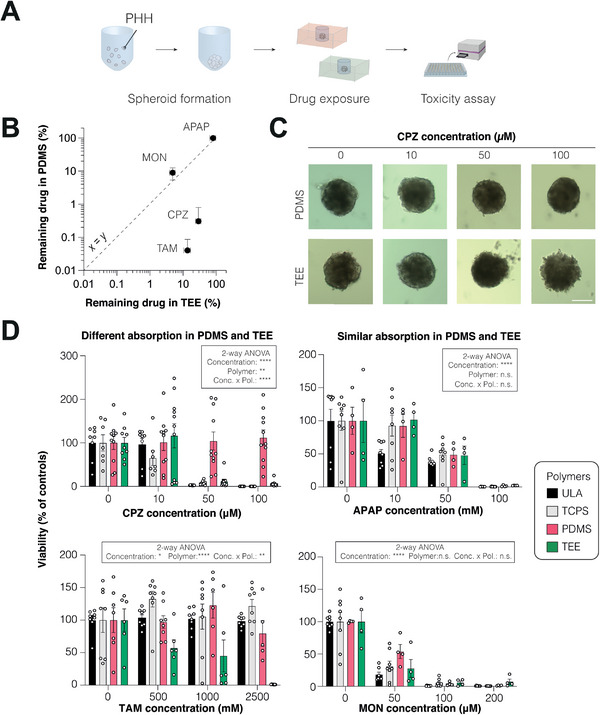
Drug absorption has major impacts on the outcomes of drug safety assessments. A) Liver spheroids comprised of primary human hepatocytes (PHH) were exposed to different doses of hepatotoxic drugs in either highly absorbing polydimethylsiloxane (PDMS) or lowly absorbing thiol‐ene epoxy (TEE) devices with otherwise identical designs. B) Scatter plot showing differences in absorption of the test substances between PDMS and TEE. Note that chlorpromazine (CPZ) and tamoxifen (TAM) absorption differ in absorption by multiple orders of magnitude between polymers whereas absorption was not significantly different for acetaminophen (APAP) and montelukast (MON). Absorption is shown as mean ± SEM for each compound from three independent TEE (*x*‐axis) and PDMS (*y*‐axis) devices. C) Representative brightfield images of liver spheroids exposed to different concentrations of CPZ. Note that spheroids in TEE devices shed cells and show loss of spheroid integrity at higher concentrations (50–100 µm) indicative of cellular toxicity. In contrast, spheroids in PDMS devices retain their spheroidal architecture with intact spheroid borders. D) Quantitative assessments of spheroid viability were performed in PDMS (red columns) and TEE devices (green columns), with ULA (black columns) and TCPS (grey columns) plates as assay references. Notably, for CPZ and TAM toxicity significantly differed between devices in agreement with stark differences in absorption patterns. In contrast, compounds with indistinguishable absorption (APAP and MON) did not show divergent toxicity profiles. Data are shown as mean ± SEM (*n* = 4–11 biological replicates from three different PHH donors). *P*‐values in boxes refer to results from two‐way ANOVA. *P < 0.05, **P < 0.01, ***P < 0.001, ****P < 0.0001. APAP = acetaminophen; CPZ = chlorpromazine; MON = montelukast; PDMS = polydimethylsiloxane; TAM = tamoxifen; TCPS = tissue culture polystyrene, TEE = thiol‐ene epoxy, ULA = ultralow attachment plate.

To directly assess the extent of small molecule absorption on the outcomes of preclinical safety test, we evaluated the hepatotoxicity of highly and lowly absorbing drugs in organotypic 3D liver cultures (**Figure** **4**A). Specifically, we tested chlorpromazine and tamoxifen as compounds that differed in absorption between PDMS and TEE, as well as montelukast and acetaminophen whose absorption was not significantly different between the materials (Figure 4B).

Importantly, human liver spheroids cultured exposed to increasing concentrations of the hepatotoxin chlorpromazine in PDMS devices did not exhibit visible changes in morphology or reduction in cell viability even at the highest concentrations tested (Figure 4C,D). In contrast, chlorpromazine toxicity was detected at therapeutically relevant concentrations in lowly absorbing TEE devices (115.1% vs 5.9% viability in PDMS vs TEE, *P* < 0.001). Similarly, the toxicity of tamoxifen was significantly underestimated in PDMS (89.4% vs 1.0% viability in PDMS vs TEE, *P* < 0.05). In contrast, both acetaminophen and montelukast showed indistinguishable dose responses, consistent with their similar absorption profiles in PDMS and TEE devices. Toxicity of acetaminophen, chlorpromazine, and montelukast was similar in TEE devices and conventionally used standard assay formats, such as ULA or TCPS plates with identical fill volumes, except for tamoxifen for which toxicity was only detected in TEE devices. These data thus demonstrate that drug absorption of cell culture polymers can skew preclinical safety assessments and contribute to potentially dangerous underprediction of compound toxicity.

### 2.4 Correcting for Drug Absorption Improves the Concordance with Clinical Toxicity Data

Next, we evaluated whether correction for absorption could improve the alignment of in vitro toxicity assessments with clinical safety data (**Table** **2**). To this end, we used the measured absorption indices after 24 h, as longer incubations resulted in only, if at all, minor differences (Figure S5, Supporting Information). Furthermore, 24 h constitutes an often‐used timepoint for medium exchanges or the measurement of experimental endpoint of toxicity, thus facilitating comparability with the published literature. In TEE, the half‐maximal toxic concentration (TC_50_) of chlorpromazine was between 10 and 50 µm after 24 h exposure. Considering its absorption rate at 37 °C (93%), the TC_50_ of free chlorpromazine is 0.7–4.5 µm, which is in excellent agreement with clinically reported toxic concentrations in patient plasma (1.5–6 µm).^[^ ^37^ ^]^ Similarly, good concordance with human toxicity data was also observed for montelukast and acetaminophen. When correcting for absorption, the TC_50_ of montelukast was 0.4–2 µm with reported toxic concentrations in patients of ≈1 µm.^[^ ^38^ ^]^ Similarly, acetaminophen toxicity was detected at 10 mm, while plasma concentrations of 2–4 mm are associated with liver failure due to acetaminophen overdose.^[^ ^39^ ^]^ Overall, these data corroborate that discrepancies in toxicity testing outcomes can, at least in part, be attributed to differential drug absorption and that correction for this phenomenon results in more accurate estimations of human liver safety.

**Table 2:** Correction for compound absorption resulted in good prediction of clinical liver safety. TC_50_ = concentration resulting in 50% toxicity.

Drugs	Nominal in vitro TC_50_	Absorption	Corrected in vitro TC_50_	Clinical toxic plasma concentrations
Chlorpromazine	10–50 µm	93%	0.7–4.5 µm	1.5–6 µm
Montelukast	10–50 µm	96%	0.4–2 µm	1 µm
Acetaminophen	≈10 mm	0%	≈10 mm	2–4 mm
Tamoxifen	500 µm	86%	75 µm	No available data

### 2.5 Compound Hydrophobicity is the Major Predictor of Drug Absorption

**Figure 5:**
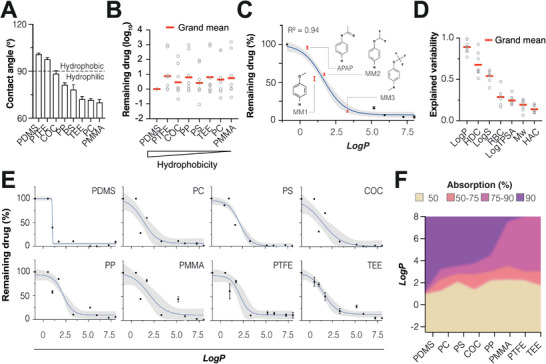
Compound hydrophobicity and the number of hydrogen donors are the main predictors of drug absorption. A) Contact angle measurements to determine the surface energy of the different polymers. Data is shown as mean ± SEM (*n* = 6 independent measurements). B) Polymer hydrophobicity does not correlate with drug absorption. Remaining drug concentration in a given polymer was expressed relative to PDMS. For clarity, averages from three independent devices are shown for each drug. C) Compound *logP* shows a highly significant association with drug absorption across all polymers tested (*R* ^2^ = 0.94; *P* < 0.0001). The blue line represents sigmoidal fit. The 95% confidence band is shown in gray. APAP and its chemical derivates with increased *logP* are highlighted. D) Besides *logP*, hydrogen donor count (HDC), *logS* and the rotatable bond count (RBC) correlated significantly with compound absorption. E) While *logP* clearly correlated with absorption in all polymers, the shape of the association differed drastically between polymers. Data is shown as mean ± SEM for each compound measurement from three independent polymer devices. The blue line represents sigmoidal fit. The 95% confidence band is shown in grey. F) 2D summary of absorption data for all compounds and polymers. Note that while none of the polymers absorb compounds with *logP* < 1, pronounced differences in absorption are apparent for compounds with *logP* ≥ 2. AMI = amiodarone; APAP = acetaminophen; COC = cyclic olefin co‐polymer, CPZ = chlorpromazine; MM1‐3 = APAP derivates 1–3; MON = montelukast; MTX = methotrexate; PC = polycarbonate; PDMS = polydimethylsiloxane; PMMA = polymethylmethacrylate; PP = polypropylene; PS = polystyrene; PTFE = polytetrafluoroethylene; TAM = tamoxifen; TEE = thiol‐ene epoxy.

To understand whether compound, polymer properties or their interaction drive absorption, we first evaluated the surface energy of the different materials. Water contact angles of the polymers differed between 100.8° for PDMS (most hydrophobic) to around 70° for PMMA, PC, and TEE (most hydrophilic; **Figure** **5**A). Notably, no association was observed between absorption and material surface hydrophobicity (*R* ^2^ = 0.007; *P* = 0.5; Figure 5B).

In contrast to the lack of association with polymer hydrophobicity, we found that compound hydrophobicity correlated well with absorption in agreement with previous studies in PDMS.^[^ ^40^ ^]^ Specifically, we found that the hydrophobic compounds amiodarone, montelukast, tamoxifen, and chlorpromazine (all with *logP* > 5) exhibited significant absorption across polymers, whereas the hydrophilic drugs acetaminophen and methotrexate (both with *logP* < 1) were nonabsorptive. To functionally test this correlation, we employed a chemical tuning strategy in which we used acetaminophen derivates (MM1‐MM3) with increasing hydrophobicity (*logP* of 1.01, 1.7, and 3.32, respectively). Indeed, as expected, these compounds featured drastically increased absorption despite their structural similarity to nonabsorptive acetaminophen. Overall, absorption was well approximated by a sigmoidal association with compound *logP* (*R* ^2^ = 0.94; Figure 5C; and Figure S6, Supporting Information). By comparison, other measures of compound hydrophilicity, such as *logS*, were weaker overall predictors of compound absorption (*R* ^2^ = 0.58; Figure 5D; and Table S3, Supporting Information). Besides hydrophobicity the number of hydrogen donors (*R* ^2^ = 0.73) and the number of rotational bonds (*R* ^2^ = 0.28) were decent predictors of absorption, whereas the number of hydrogen acceptors, molecular weight or topological polar surface area (TPSA) did not associate with absorption. These results demonstrate that compound hydrophobicity is the main determinant of drug absorption in polymers.

Interestingly, while absorption increased with *logP* across all tested polymers, the materials exhibited pronounced differences in *logP* sensitivity (Figure 5E). PDMS exhibited an almost binary absorption profile with negligible absorption for compounds with *logP* < 1 and absorption >90% for *logP* > 1.25. In contrast, associations were considerably less steep and overall absorption of hydrophobic compounds less pronounced for PMMA, PTFE, and TEE. On the basis of these data, we conclude that compound absorption to polymers can be disregarded for hydrophilic compounds with *logP* < 1 irrespective of platform polymer (Figure 5F). For more hydrophobic compounds, drastic differences in absorption profiles can be observed. Compounds with *logP* values between 2 and 3.5 absorbed similarly in all polymers except for PDMS for which absorption is significantly higher. For highly hydrophobic compounds with *logP* > 3.5, PTFE and TEE were the least absorptive. Combined, these results identify the critical determinants of compound absorption and provide important guidance for the selection of platform material for pharmacological and toxicological assessments.

## 3 Discussion

Absorption to cell culture devices has long been recognized as an important limitation for studies involving small molecules. Using the lipophilic dye Nile Red, initial seminal work demonstrated absorption into the bulks of PDMS microdevices and concluded with the suggestion to re‐evaluate the effective dose of test compounds used in screenings.^[^ ^29^ ^]^ Absorption of compounds is dependent on device design parameters, such as volume‐to‐surface ratio, temperature, and solution parameters, such as viscosity, salinity, and pH. However, the heterogeneity of previous studies hampered the meaningful meta‐analysis of preexisting data. We here fabricated devices with identical designs and used the same freshly prepared solutions at identical temperatures to eliminate these variables and allow well‐controlled *ceteris paribus* conclusions about the differences in absorption between polymers and compounds. Our computational and experimental results are consistent with prior studies in PDMS that reported that compound hydrophobicity constitutes the main driver of absorption, particularly for compounds with *logP* > 1.85 and low hydrogen donor numbers.^[^ ^41^ ^]^ While hydrophilic compounds such as methotrexate are not absorbed to any platform material in agreement with previous reports,^[^ ^42^ ^]^ hydrophobic compounds are absorbed in all polymers albeit to variable extents. In contrast to compound hydrophobicity, TPSA was only weakly correlated. These results contrast previous reports, possibly due to the very small number of compounds (*n* = 4) analyzed.^[^ ^30^ ^]^

Multiple analyses in thermoplastics found that absorption was mostly explained by acidity and basicity rather than compound hydrophobicity.^[^ ^43^, ^44^ ^]^ However, these studies evaluated absorption after relatively short periods of time (2–4.5 h), which could explain the differences in the identified key parameters. It was previously shown in PS devices that absorption exhibits a biphasic profile in which rapid compound partitioning can be observed during the first hours and then slows down.^[^ ^45^ ^]^ This profile was hypothesized to be due to the initial rapid adsorption of the compound to the surface of the device followed by slower diffusion within the polymer bulk. Notably, the relative importance of adsorption and absorption for explaining the loss of compound likely differs between polymers and the timeline of the respective experiment. This is corroborated by our numerical modeling, which well approximated our experimental results in PDMS, but overpredicted absorption in glassy polymers, an effect that could not be explained by different in‐polymer diffusion coefficients. While our study was not geared to distinguish between adsorption and absorption, it can thus be assumed that absorption will be most important for elastomers, whereas adsorption might be most dominant in glassy thermosets and thermoplastics.

In contrast to compound hydrophobicity, the hydrophobicity of the platform material did not significantly correlate with drug absorption. Yet, the extent of absorption differed by up to three orders of magnitude between materials with PDMS being the most absorptive and least absorption observed in PTFE and TEE. Overall, interactions of small molecules and polymers are primarily driven by hydrophobic interactions and pore‐filling effects related to material porosity.^[^ ^46^ ^]^ Pore‐filling effects can exist in both glassy (COC, PC, PMMA, PP, PS, PTFE, and TEE) and rubbery polymers (PDMS), but the inherent porosity of PDMS can explain the excessive absorption compared to other polymers. In addition, the rubbery state of PDMS resulting from its macromolecular structure with linear randomly arranged polymer chains, in conjunction with its high hydrophobicity allows solvent to enter the interstitial space between polymer chains leading to a positive feedback of swelling and small compound absorption.^[^ ^47^, ^48^ ^]^ While PTFE is similarly hydrophobic, the highly electronegative fluorine atoms prevent water and soluble small compounds from entering the polymer bulk, thereby reducing absorption despite its hydrophobic surface.^[^ ^49^ ^]^

Besides differences in polymer physicochemical properties, differences in surface roughness could impact absorption. Notably, we minimized surface roughness in the fabrication process flow of micromilled devices and owing to the high‐fidelity replication in casting of PDMS and TEE on the aluminum molds,^[^ ^50^ ^]^ we expect the same surface roughness and consequently similar surface area for both replicated and directly milled parts. However, minor effects due to the different fabrication techniques cannot be excluded.

Absorption differences between polymers can have important effects on the outcomes of pharmacological and toxicological assessments. As we demonstrated here, in highly absorptive PDMS devices, chlorpromazine toxicity was grossly underestimated even at the highest dose tested and would have been falsely classified as nonhepatotoxic in preclinical safety assessments. However, significant toxicity was apparent when isogenic liver spheroid cultures were exposed in TEE devices with lower absorption. Notably, while we analyzed the loss of compound in devices that resemble wells in standard 96‐well plates to facilitate comparability, further factors need to be considered in MPS. By design, we can assume that absorption will be amplified in MPS, as the addition of microfluidic channels typically results in lower volume‐to‐surface ratios.^[^ ^51^ ^]^ In single‐pass devices without circulation, this effect is however counteracted by perfusion, as the constant replenishment with “fresh” compound can compensate for absorption given that flow rates are sufficiently high. Furthermore, additional parameters, such as the choice of peristaltic tubing, constitute relevant determinants of absorption that need to be considered in MPS.^[^ ^52^ ^]^ Correcting for in vitro for drug absorption, resulted in better alignment between toxic concentrations and clinically relevant plasma concentrations in the static system as shown here. However, further studies will be required to evaluate the effect of perfusion on saturation kinetics and biological outcomes in MPS.

Different strategies can be employed to minimize absorption. Fabrication of devices from less absorptive materials has been shown to directly impact the results of pharmacological and toxicological assays. For instance, culture of hepatic cell lines or primary hepatocytes in tetrafluoroethylene‐propylene or glass‐based devices reduced absorption of small hydrophobic molecules, increased the amount of detectable metabolites and sensitized 2D hepatocyte cultures to coumarin cytotoxicity.^[^ ^53^, ^54^ ^]^ However, there are typically tradeoffs for the use of materials with decreased compound absorption, such as unfavorable mechanical or optical properties, reduced ease of prototyping or higher fabrication costs.

Alternatively, absorption can be reduced by treatment with oxygen plasma, UV/ozone, different surface modifications and coating strategies.^[^ ^55^ ^]^ Promising approaches that were shown to reduce compound absorption of PDMS devices include surface coating with poly(ethylene glycol) (PEG),^[^ ^56^ ^]^ sol–gel treatment,^[^ ^57^ ^]^ PTFE coating,^[^ ^58^ ^]^ the use of PEG‐PDMS block copolymers,^[^ ^59^ ^]^ incorporation of poly(ethylene oxide) silane amphiphile (PEO‐SA) into the silicon matrix^[^ ^60^ ^]^ or the dip‐coating in macrocyclic polyphenols.^[^ ^61^ ^]^ While these and other treatments can reduce compound absorption, they also create undesired complexities that reduce throughput and may alter device properties, such as stiffness or optical transparency. Moreover, surface treatments are often not durable which can lead to their delamination rendering them unsuitable for long‐term applications. Evaluations of coating approaches are mostly limited to PDMS, and it will be interesting to evaluate the performance of these strategies in reducing the partitioning of hydrophobic compounds in materials with lower baseline absorption. Furthermore, absorption can be impacted by the composition of the solution, particularly regarding protein concentrations. Here, we used serum‐free culture conditions and, as albumin secretion of spheroids is <1 pg cell^−1^ h^−1^,^[^ ^62^ ^]^ protein concentrations in the medium can be considered to be negligible.

Besides technical attempts to minimize absorption, there are also computational strategies to correct for absorption in cell culture experiments. While many physiologically based pharmacokinetic (PBPK) and in vitro–in vivo extrapolation (IVIVE) models neglect compound absorption to culture vessels, absorption into the polymer has been acknowledged as a factor that can reduce the concentrations of free compound in the in vitro model and impact toxicological evaluations.^[^ ^63^, ^64^ ^]^ Specifically, experimentally determined medium‐plastic partition coefficients can be factored into multiple compartment models to account for material shortcomings.^[^ ^65^, ^66^, ^67^ ^]^ However, most models are currently limited to specific chemical classes or polymers. As such, the absorption data provided here can serve as a useful resource to inform more generalizable computational approaches for polymers beyond polystyrene and PDMS.

Previous analyses of 800 oral drugs from multiple pharmaceutical companies demonstrated that *logP* did not associate with failure rates in preclinical development, whereas there was a significant overrepresentation of hydrophobic compounds that failed in clinical trials due to safety.^[^ ^68^ ^]^ Our data would suggest that at least part of the false negative preclinical safety assessments could be attributed to excessive absorption of hydrophobic compounds in conventional in vitro toxicity assays. Thus, in light of the current drug development trends that favor increasing proportions of hydrophobic drugs over the last 25 years^[^ ^69^, ^70^ ^]^ and the resourceintensive process of lead compound modifications, selecting polymers with lower absorption across a wider *logP* range can be expected to improve the translatability of pharmacological and toxicological in vitro assessments.

In conclusion, the presented study quantifies drug absorption across different cell culture polymers and identifies key compound parameters that govern absorption. Absorption patterns differed between polymers by multiple orders of magnitude with PDMS being the most absorptive, whereas PTFE and TEE exhibited the overall lowest absorption, particularly for compounds with *logP*>3.5. We moreover demonstrate that consideration of compound absorption by selecting lowly absorbing platform materials or post hoc correction is of paramount importance for the development of predictive MPS with improved preclinical accuracy.

## 4 Experimental Section

### Chemicals

The following chemicals were purchased from Sigma‐Aldrich (Sweden) with purity ≥98%: amiodarone hydrochloride (abbreviated henceforth as AMI; CAS no. 19774‐82‐4), chlorpromazine hydrochloride (CPZ; CAS no. 69‐09‐0), acetaminophen (APAP; CAS no. 103‐90‐2), 4‐methoxy‐aniline (MM1; CAS no. 104‐94‐9), 4‐(difluoromethoxy)‐aniline (MM2; CAS no. 22236‐10‐8), *N*‐methyl‐4‐(trifluoromethoxy)‐aniline (MM3; CAS no. 41419‐59‐4), montelukast sodium (MON; CAS no. 151767‐02‐1), tamoxifen (TAM; CAS no. 10540‐29‐1), and methotrexate trihydrate (MTX; CAS no. 59‐05‐02). Pruvanserine (CAS no. 443144‐26‐1) was obtained from Merck (Darmstadt, Germany).

### Fabrication of Polymer Devices

Polymer devices of COC, PS, PC, PP, PMMA, and PTFE polymers were fabricated via CNC precision micromilling using a MiniMill GX (Minitech Machinery Corp., USA). In the first step, devices were milled through an initial coarse run with a higher feed rate and bigger z‐steps leaving 100 µm, followed by a second fine‐milling run with a lower feed rate and smaller z‐steps. PDMS and TEE devices were fabricated via casting. The devices were comprised of wells with a diameter of 6 mm, depth of 3.2 mm, and pitch of 18 mm (Figure S1, Supporting Information). For PDMS and TEE polymers, the casting of polymer precursors was implemented for the replication of the devices. Aluminum molds were micromilled using the same milling machine as described above. Aluminum molds featured a square cavity with an array of 16 pillars with a diameter of 6 mm, depth of 3.3 mm, and pitch of 18 mm. To facilitate the demolding process, the surface of the aluminum molds was teflonized. To this end, Teflon AF 1601 (Chemours, USA) was diluted 1:6 with fluorinert FC‐40 (Sigma‐Aldrich, Germany) and the Teflon solution was then cast on the aluminum molds, followed by baking at 180 °C for 10 min. Next, TEE resin (OSTEMER 322, Sweden) and PDMS polymer precursors (Sylgard 184, Dow Corning, USA) were mixed and degassed separately. Next to that, each polymer resin was cast in an aluminum mold. TEE resin was exposed to collimated UV‐light (14 mW cm^−2^) for 120 s and then thermally cured at 70 °C overnight. PDMS was only subject to thermal curing at 70 °C for 1 h. After curing, TEE and PDMS polymers were demolded.

### Contact Angle Measurement

Contact angle (CA) measurements were performed using a Theta Lite optical tensiometer (TL100, Finland) on all the polymers selected in this study. Six random regions on each sample were chosen and the average CA were calculated.

### Chemical Feature Predictions

*LogP* of each compound was predicted using MiLogP v.2.2 (www.molinspiration.com), AlogPS v2.1,^[^ ^71^ ^]^ MolGpKa.^[^ ^72^ ^]^ Other chemical features were extracted from PubChem (as of Jan 8, 2023).

### Absorption Measurement

For each compound, 10 mm stock solution was prepared by dissolving an appropriate amount of the compound in dimethyl sulfoxide (DMSO) in disposable amber glass vials. 1 µm working solutions were prepared by diluting respective stocks (1:10 000) in Dulbecco's phosphate buffered saline (PBS) pH 7.5. An equal amount of the working solutions (60 µL) was pipetted into wells in the polymer devices (3 independent replicates for each time point in each polymer device) and incubated at room temperature (RT) or 37 °C as indicated. Unless stated otherwise, at either 2 or 24 h, the solutions were gently mixed with pipettes and 10 µL of the solutions was mixed with 20 µL of chilled stop solutions. The stop solution comprises of 99% acetonitrile (ACN), 1% formic acid, with either 0.03% or 0.003% 10 mm pruvanserine. In addition, 10 µL of working solutions of each drug was sampled at the start of the experiment as a baseline (concentration at *t*_0_).

### Liquid Chromatography with Tandem Mass Spectroscopy (Lc‐Ms/Ms) Analysis

LC‐MS/MS was performed with an AB Sciex API 6500+ triple quadrupole (AB Sciex LLC, MA) coupled with a Waters Acquity I‐Class ultraperformance liquid chromatography (UPLC) (Waters Corporation, MA). The software Analyst v1.7 (AB Sciex LLC) was used for control. An Acquity UPLC BEH C18 1.7 µm, 2.1 mm × 50 mm column (AB Sciex LLC) was applied. As mobile phase A, 70 mm ammonium formate buffer containing 0.1% v/v formic acid and as mobile phase B, ACN was used. The injection volume was 4 µL. The gradient conditions were the following: 0–0.1 min 0% B, 0.1–0.8 min 0–100% B, 0.8–1.0 min 100% B, 1.0–1.4 min 0% B. The flow rate was 0.8 mL min^−1^ and data collection occurred between 0.3 and 1.7 min. Electrospray ionization in positive mode with multiple reaction monitoring was used. The dwell time for each transition was 20 ms. The parameter settings are summarized in Table S1 (Supporting Information).

Free drug concentrations were determined from the area under the curve (AUC) of the product ion spectra and normalized to the AUC of pruvanserine. Absorption (%) was calculated according to Equation 1.

(1) $$\begin{array}{c} \mathrm{Absorption}\left(\%\right)=\left(\frac{C_{0}-C_{t}}{C_{0}}\right)\times 100=100-\mathrm{remaining}\;\mathrm{drug}\;\left(\%\right) \end{array}$$

with *C*_0_ as compound concentration at *t*_0_ and *C*_t_ as compound concentration at the indicated time point *t*, respectively.

### Absorption Index of Polymers

To rank the polymer performance based on their absorptive capacity, the absorption index (*AI*) was defined as a partition coefficient of a compound between polymer device and solutions (DPBS pH 7.5) as defined in (Equation (2))

(2) $$\begin{array}{ccc} \mathrm{LogAI} & = & \log\left(\frac{\mathrm{Amount}\;\mathrm{of}\;\mathrm{compound}\;\mathrm{in}\;\mathrm{polymer}}{\mathrm{Amount}\;\mathrm{of}\;\mathrm{compound}\;\mathrm{in}\;\mathrm{solution}}\right)\\ & = & \log\left(\frac{\mathrm{Absorbed}\;\mathrm{compound}\left(\%\right)}{\mathrm{Remaining}\;\mathrm{compound}\left(\%\right)}\right) \end{array}$$

### Cell Culture and Toxicity Testing

Cryopreserved PHH from three donors (Table S2, Supporting Information) were seeded and formed as previously reported.^[^ ^15^ ^]^ Informed consent from each donor or the subject's legally authorized representative was retrieved by the supplier in accordance with HHS regulations for the protection of human subjects (45 CFR §46.116 and §46.117) and Good Clinical Practice (ICH E6). After formation, PHH spheroids were cultured in William's E medium supplemented with 100 nm dexamethasone, 1.7 µm insulin, 11 mm glucose, 2 mm L‐glutamine, 5.5 µg mL^−1^ transferrin, 6.7 ng mL^−1^ sodium selenite, 100 U mL^−1^ penicillin, and 100 µg mL^−1^ streptomycin. Prior to toxicity screening, the devices were disinfected with 70% ethanol and air‐dried for at least 30 min. As a reference, ultralow attachment (ULA) plates (Corning, USA) and tissue‐culture polystyrene (TCPS; VWR, Sweden) plates were used. Prewarmed culture medium containing the indicated compound concentrations was dispensed into each polymer device. Subsequently, PHH spheroids were loaded and the device was immediately covered. Each device was maintained in 37 °C incubator (5% CO_2_) in a humidity chamber to minimize evaporation. Following 24 h of compound exposure, cell viability was measured by ATP quantification (CellTiter Glo; Promega, USA) and normalized to vehicle treated controls.

### Computational Simulation of Drug Absorption

A finite element simulation was constructed to analyze drug concentration within a 3D cylindrical geometry with a diameter of 6 mm and a height of 2.12 mm (corresponding to the height of 60 µL solution in the well) surrounded by 6 mm of polymer on the side and 3 mm at the bottom using COMSOL Multiphysics 6.1 (COMSOL Inc., USA). The model was built under the following assumptions, in alignment with the mass transport literature^[^ ^73^, ^74^ ^]^: 1) instantaneous equilibrium between the polymer and drug solution at the interface, 2) the equilibrium distribution of the drug between the two phases is linear and is characterized by the partition coefficient (P); 3) the value of P remains constant, particularly within the context of the low concentrations employed in the study. Fick's second law of diffusion served as the foundation for modeling mass transport within both the solution (Equation (3)) and the polymer (Equation (4))

(3) $$\frac{\partial c_{s}}{\partial t}-\nabla .\left(D_{s}.\;\nabla c_{s}\right)=0$$

(4) $$\frac{\partial c_{p}}{\partial t}-\nabla .\;\left(D_{\mathrm{polymer}}.\;\nabla c_{p}\right)=0$$

Here, *D*_s_ represents the diffusion coefficient of the drug within the solution, while *D*_polymer_ stands for the diffusion coefficient of the drug within the polymer. The variables *c*_s_ and *c*_p_ denote the concentrations of the drug within the solution and polymer, respectively. The assumption of equilibrium at the solution‐polymer interface yields the boundary condition given in (Equation (5))

(5) $$C_{i,p}=P\times C_{i,s}$$

where *C*_i,p_ represents the interfacial concentration of the drug on the polymer side, *C*_i,s_ corresponds to the interfacial concentration of the drug on the solution side, and *P* is the partition coefficient. In order to calculate the fractional surface coverage (adsorption) of the compounds, the Langmuir isotherm was adopted according to (Equation (6))

(6) $$\theta =\frac{K_{\mathrm{ads}}\times c_{A}}{1+K_{\mathrm{ads}}\times c_{A}}$$

Where *θ* is the partial coverage of adsorbate, *K*_ads_ is equilibrium constant for adsorption, and *c*_A_ is the concentration of compound at the surface in the adjacent bulk phase. The influence of varying *D*_polymer_ and the partition coefficient on the drug's concentration within the well over 24 h was explored. For PDMS, the diffusion coefficient of each drug was estimated utilizing a scaling relation previously documented for small molecules,^[^ ^75^, ^76^ ^]^ resulting in a spectrum of diffusion coefficients ranging from 10^−13^ to 10^−12^ m^2^ s^−1^ in line with previous reports.^[^ ^77^ ^]^ For glassy polymers, diffusion coefficients from 10^−18^ to 10^−15^ m^2^ s^−1^ were considered.^[^ ^78^ ^]^ To refine accuracy near the polymer‐solution interfaces, a customized meshing sequence was employed.

### Statistical Analysis

Results are presented as mean ± SEM unless stated otherwise. Statistical tests were conducted using Prism (Version 9, GraphPad, USA). For determining statistical significance between two groups, unpaired two‐tailed heteroscedastic *t*‐tests were performed with *n* ≥ 3 samples per group. Differences in toxicity between polymers and exposure concentrations were evaluated using 2‐way ANOVA with *n* ≥ 4 samples per group. All data are shown, and no outlier removal was performed. For all results, significance was defined as *P* ≤ 0.05. Multiple testing was accounted for using Benjamini–Hochberg correction with a false discovery rate (FDR) of 10%.

## Conflict of Interest

VML is CEO and shareholder of HepaPredict AB, as well as co‐founder and shareholder of PersoMedix AB. AMK, RZS and VML have applied for a patent pertaining to thiolene devices. The other authors do not disclose competing interests.

## Author Contributions

A.M.K. and R.Z.S. contributed equally to this work. A.M.K. performed drug exposure experiments, generated the illustrations and figures, and wrote the first draft; R.Z.S. fabricated the polymer devices; N.T. conducted COMSOL modeling of drug partitioning; M.M. contributed acetaminophen derivates; L.P. and U.H. performed LC‐MS/MS measurements. R.Z.S. and V.M.L. conceived the study. V.M.L. supervised the study. All authors contributed to data analysis and manuscript revision.

## Supporting information

Supporting Information

Supporting Information

## Data Availability

The data that support the findings of this study are available from the corresponding author upon reasonable request.
