# Supplementary material for: Compound Absorption in Polymer Devices Impairs the Translatability of Preclinical Safety Assessments
Source: Adv Healthc Mater. 2023 Dec 10;13(11):2303561. doi: 10.1002/adhm.202303561 (PMC11469150; doi:10.1002/adhm.202303561)
Supplement: Supplementary file 1 — Supporting Information [file ADHM-13-2303561-s001.pdf]

ADVANCED HEALTHCARE MATERIALS

Supporting Information

for *Adv. Healthcare Mater.*, DOI 10.1002/adhm.202303561

Compound Absorption in Polymer Devices Impairs the Translatability of Preclinical Safety Assessments

Aurino M. Kemas, Reza Zandi Shafagh, Nayere Taebnia, Maurice Michel, Lena Preiss, Ute Hofmann and Volker M. Lauschke**

Supplementary Table 1. **Mass spectrometry settings for the LC-MS/MS system.** *DP* = declustering potential; *CE* = collision energy; *CXP* = cell exit potential

**Compounds**	**Q1 mass (Da)**	**Q3 mass (Da)**	**DP (volts)**	**CE (volts)**	**CXP (volts)**
Pruvanserin (internal standard)	377.2	209.1	59	32	11
Acetaminophen, APAP	152.1	110.0	57	23	5
Amiodarone, AMI	646.2	58.1	110	90	9
Chlorpromazine, CPZ	319.1	86.2	47	29	3
Methotrexate, MTX	455.1	307.9	56	29	20
Montelukast, MON	586.1	421.9	61	37	12
Tamoxifen, TAM	372.2	72. 0	96	47	4
APAP derivate 1, MM1	124.07	92.96	80	25	17
APAP derivate 2, MM2	192.13	108.02	84	40	11
APAP derivate 3, MM3	160.07	110.07	89	28	11

Supplementary Table 2. Information of the utilized hepatocyte donors.

Donor ID	1	2	3
Sex	Female	Female	Male
Age	30	27	25
Ethnicity	Hispanic	African American	Hispanic
BMI	30.8	28.2	32.2
Cause of death	Head trauma	Anoxia, respiratory disease	Head trauma
Drugs	Opiate dependency	None	Smoke marijuana daily
Alcohol	1-2 beers per day socially	None	6 beers and tequilla 2 times per month past 7 years
Tobacco	1-2 cigarettes per day for 14+ years	None	1-5 cigarettes per week past 8 years

Supplementary Table 3. Regression analysis of different chemical features versus compound absorption.

Independent variables	Predictor algorithm	Explained variability, R^2	
		Sigmoidal	Linear
LogP	AlogPS v.2.1	0.94	0.83
LogS	AlogPS v.2.1	0.58	0.40
LogTPSA	AlogPS v.2.1	n.s.	0.27
Hydrogen donor count	ChemAxon	0.73	0.51
Hydrogen acceptor count	ChemAxon	n.s.	0.15
Rotatable bond count	ChemAxon	0.28	0.16
Molecular weight	-	n.s.	0.20

n.s. = not significant
