# Supplementary figures and images for: Compound Absorption in Polymer Devices Impairs the Translatability of Preclinical Safety Assessments
Source: Adv Healthc Mater. 2023 Dec 10;13(11):2303561. doi: 10.1002/adhm.202303561 (PMC11469150; doi:10.1002/adhm.202303561)

**A**

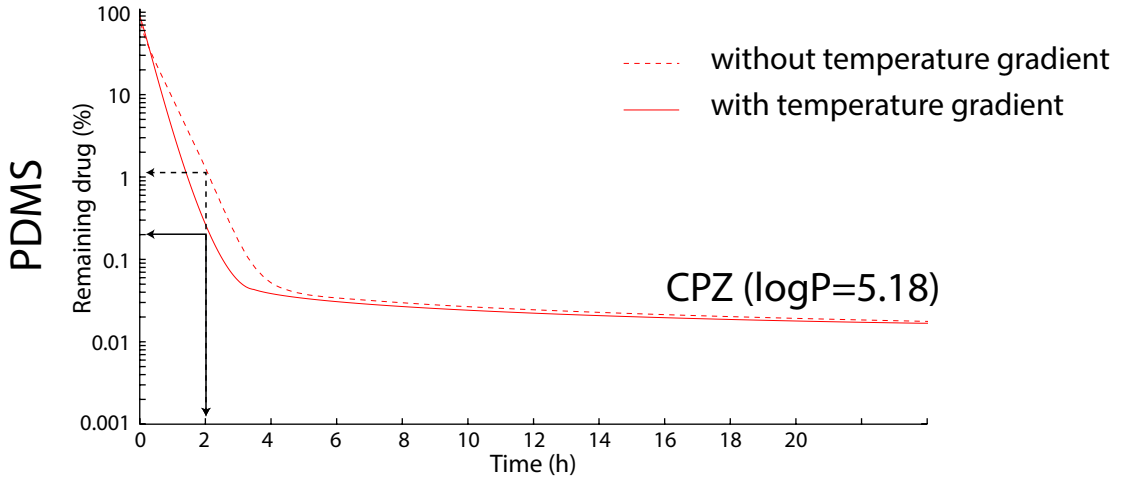

**B**

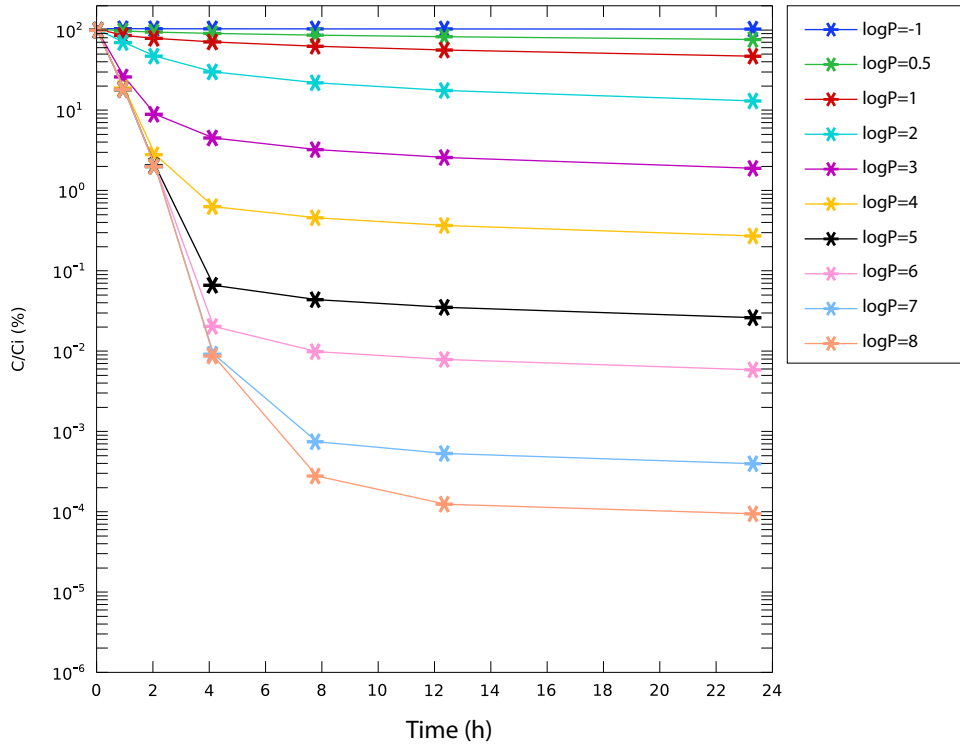

Supplement: Supplementary file 2 — Supporting Information [file ADHM-13-2303561-s002.zip › Sup Fig 4_NaTa.pdf]

# Supplementary Figure 5

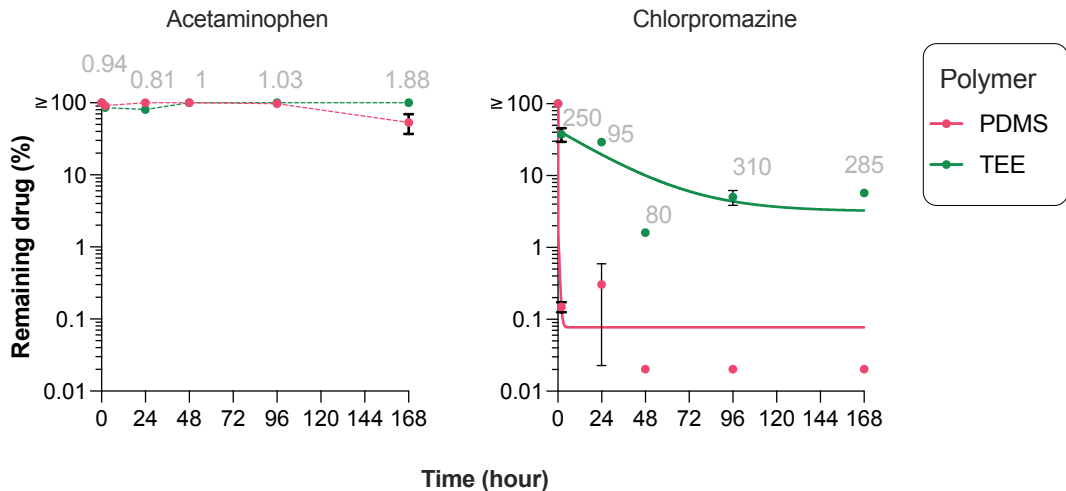

Supplement: Supplementary file 2 — Supporting Information [file ADHM-13-2303561-s002.zip › Sup Fig 5-long term incubation.pdf]

# Supplementary Figure 6

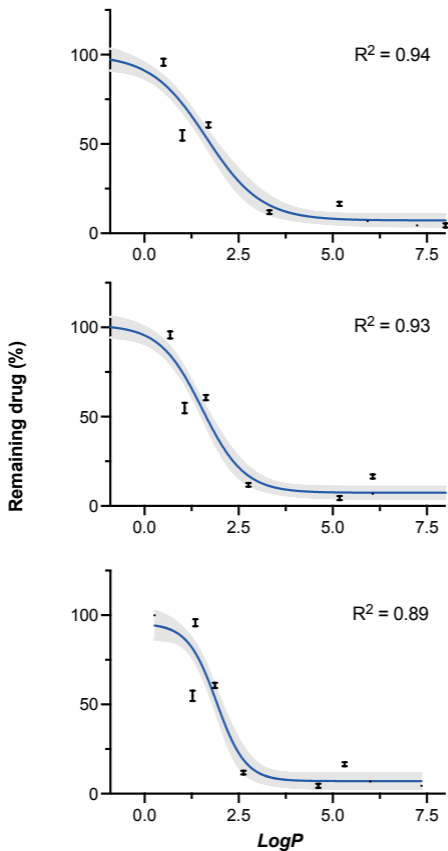

Supplement: Supplementary file 2 — Supporting Information [file ADHM-13-2303561-s002.zip › Sup Fig 6-logP determination.pdf]

# Supp. Figure 1

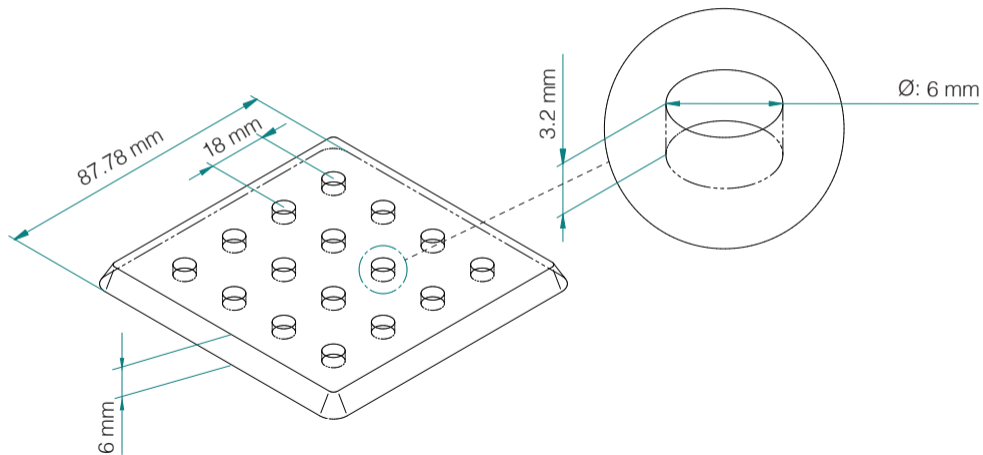

Supplement: Supplementary file 2 — Supporting Information [file ADHM-13-2303561-s002.zip › Sup. Fig 1-Dimension_corrected.pdf]

# Supp. Figure 2

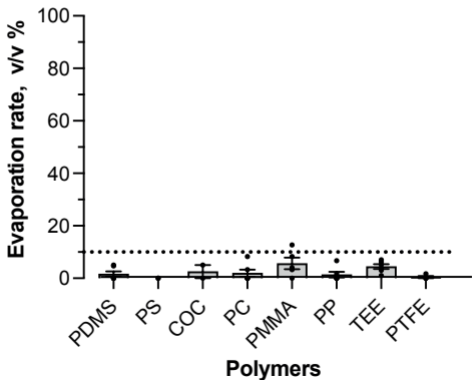

Supplement: Supplementary file 2 — Supporting Information [file ADHM-13-2303561-s002.zip › Sup. Fig 2-evaporation.pdf]

# Supp. Figure 3

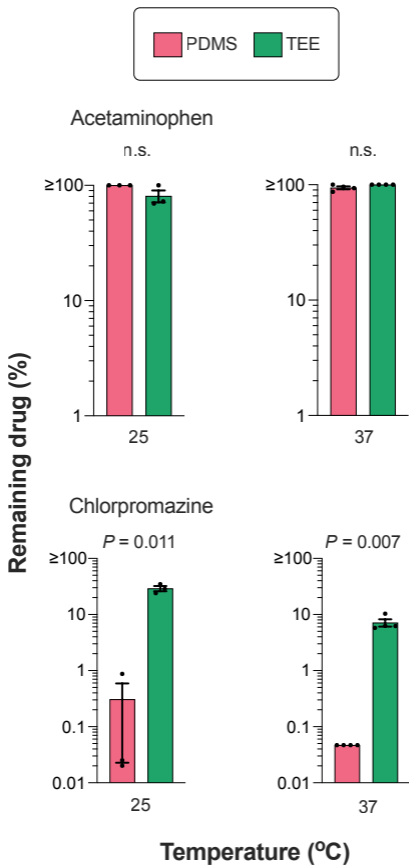

Supplement: Supplementary file 2 — Supporting Information [file ADHM-13-2303561-s002.zip › Sup. Fig 3_temperature effect.pdf]
